# Sarcopenia as a predictor of mortality in women with breast cancer: a meta-analysis and systematic review

**DOI:** 10.1186/s12885-020-6645-6

**Published:** 2020-03-04

**Authors:** Xiao-Ming Zhang, Qing-Li Dou, Yingchun Zeng, Yunzhi Yang, Andy S. K. Cheng, Wen-Wu Zhang

**Affiliations:** 1 Department of Emergency, The Affiliated Baoan Hospital of Southern Medical University, The People’s Hospital of Baoan ShenZhen, Shenzhen, Guangdong People’s Republic of China; 2 Department of Nursing, The Third Affiliated Hospital of Guangzhou Medical University, Guangzhou, China; 3 Department of Nursing, The Affiliated Baoan Hospital of Southern Medical University, The People’s Hospital of Baoan ShenZhen, Shenzhen, China; 4 Department of Rehabilitation Sciences, The Hong Kong Polytechnic University, Hong Kong, Hong Kong, China

**Keywords:** Sarcopenia, Mortality rate, Breast cancer, Systematic review and meta-analysis

## Abstract

**Background:**

Breast cancer is the most commonly diagnosed cancer and the leading cause of cancer death in women worldwide. Recently, studies have been published with inconsistent findings regarding whether sarcopenia is a risk factor for mortality in breast cancer patients. Therefore, the aim of this systematic review and meta-analysis was to systematically assess and quantify sarcopenia as a risk factor for mortality in breast cancer patients.

**Methods:**

In a systematic literature review of PubMed, EMBASE, and the Cochrane CENTRAL Library, we searched for observational studies written in English (from database inception until April 30, 2019) that reported an association between sarcopenia and breast cancer in women who were 18 years or older.

**Results:**

A total of six studies (5497 participants) were included in this meta-analysis. Breast cancer patients with sarcopenia were associated with a significantly higher risk of mortality, compared to breast cancer patients without sarcopenia (pooled HR-hazard ratio = 1.71, 95% CI: 1.25–2.33, I^2^ = 59.1%). In addition, the results of age subgroup analysis showed that participants younger than 55 years with sarcopenia had a lower risk of mortality than participants aged 55 years and older with sarcopenia (pooled HR = 1.46, 95% CI: 1.24–1.72 versus pooled HR = 1.99, 95% CI: 1.05–3.78), whereas both have an increased risk of mortality compared to non-sarcopenic patients. Subgroup analyses regarding stage at diagnosis revealed an increased risk of mortality in non-metastatic patients compared to participants without sarcopenia (pooled HR = 1.91, 95% CI: 1.32–2.78), whereas the association was not significant in metastatic breast cancer patients. Other subgroup analyses were performed using different follow-up periods (> 5 years versus ≤5 years) and the results were different (pooled HR = 1.81, 95% CI: 1.23–2.65 versus pooled HR = 1.70, 95% CI: 0.80–3.62).

**Conclusions:**

The present study found that sarcopenia is a risk factor for mortality among female early breast cancer patients. It is imperative that more research into specific interventions aimed at treating sarcopenia be conducted in the near future in order to provide evidence which could lead to decreased mortality rates in breast cancer patients.

## Background

Breast cancer is the most commonly diagnosed cancer and the leading cause of cancer death in women around the world [1]. According to global data, there were approximately 2.1 million newly diagnosed breast cancer cases in 2018, accounting for almost one in four cancer cases among women and 626,679 breast cancer deaths [2]. Although significant progress has been made in breast cancer research, it remains difficult to predict which female patients are at increased risk of short-term survival and toxicity. In addition to traditional prognostic factors (high histologic grade, lymph node status, involved margins, tumor size) [3], the identification of new clinical or biological markers is the goal of ongoing research for improving breast cancer management. Cancer patients usually suffer from changes in body composition parameters (e.g. weight loss, a typical characteristic of Cachexia). Cancer cachexia is a multidimensional syndrome that is characterized by unintended loss of both adipose tissue and lean body mass (LBM) and comes with adverse complications [4]. It is estimated that cachexia is the main cause of death among 30–50% of cancer patients [5]. In addition, lower physical function, decreased resilience to chemotherapy and radiation treatment, and generally worse prognoses are observed in cachectic patients compared to those with stable weight [6]. However, other body composition parameters including muscle quantity and density have recently become a subject of research in the field of cancer prognosis [7].

Sarcopenia is a condition defined as a syndrome associated with loss of muscle mass and strength as well as decreased physical performance in older adults [8]. It shares some characteristics with age-related changes in muscle tissue, such as decreased satellite cells and fast-twitch muscle fibers and atrophy of slow-twitch muscle fibers [9]. Numerous complex mechanisms lead to sarcopenia, including neurodegeneration, impaired signaling, inflammation, disuse, and declined nutrient intake. Sarcopenia has been shown to be prevalent in adults with cancer due to the increasing prevalence of disease with age [10]. Furthermore, inflammation and malnutrition associated with cancer may worsen muscles. Currently, there are several diagnostic imaging techniques for assessing sarcopenia including dual-energy X-ray absorptiometry (DEXA), computed tomography (CT), magnetic resonance imaging (MRI), and bioelectrical impedance analysis (BIA) [8]. Previous studies have reported that the presence of sarcopenia in patients with cancer is associated with negative clinical outcomes, such as post-operative complications [11], increased chemotherapy toxicity [12], and poorer overall survival (OS) [13]. Recently, a meta-analysis has found that sarcopenia significantly increases mortality risk among various cancer types and stages [14]. However, this study did not include breast cancer, although it is in fact the most common cancer type among women worldwide.

Inconsistent studies have been published examining whether sarcopenia is a risk factor for breast cancer mortality [15, 16]. Over the past 5 years, an increasing number of studies have reported that there is an association between sarcopenia and mortality rate among women with breast cancer [17–20]. A systematic review summarizing current literature on the evaluation of body CT-determined sarcopenia in breast cancer patients and its association with clinical outcomes has been published recently [21]. Undoubtedly, mortality is one of the most important clinical outcomes in clinical oncology. Therefore, the aim of this systematic review and meta-analysis was to systematically assess and quantify sarcopenia as a risk factor for mortality in breast cancer patients.

## Methods

We registered with the international prospective Register for Systemic Reviews for our meta-analysis with the number CRD42019138425 and conducted it according to the PRISMA guidelines.

### Search strategy and selection criteria

A systematic literature search was initially conducted by two authors independently on PubMed, EMBASE, and the Cochrane CENTRAL Library of articles dating from database inception until May 4, 2019. The search strategy combined keywords and medical subject headings (Mesh) terms, such as mortality (death, survival), breast cancer (tumor, cancer, tumour), and sarcopenia (sarcopenias, sarcopenia, presarcopenia), and was tailored to each database. We used subject terms and truncation symbols in our search strategy to find all relevant studies. In addition, when seeking potential grey literature, references to eligible articles were searched using Google. The search strategy for the PubMed database is provided as Supplementary File 1.

### Study selection

All relevant articles were examined initially (title and abstract). After that, screening was conducted independently by two blinded investigators (WWZ and YCZ). When a disagreement on study inclusion or exclusion occurred, the third reviewer (WWZ) intervened and a discussion ensued until a final consensus was reached.

### Inclusion and exclusion criteria

#### Inclusion criteria

(1) Participants: adults 18 years and over with breast cancer; (2) A clear definition of sarcopenia, defined using a consensual method: CT scan (muscle area or muscle volume or skeletal muscle index), DXA (skeletal muscle index), BIA (skeletal muscle index); (3) Design: observational study; (4) Studies exploring the association between sarcopenia and mortality among breast cancer patients.

#### Exclusion criteria

(1) Article type: only abstract, review articles, letters and laboratory research, case report; (2) Insufficient data; (3) Irrelevant outcome.

### Data extraction

The data from the selective studies were independently abstracted by two investigators (XMZ, QLD) using a standardized data-abstraction form. The following information - author, year of publication, country, demographic participant characteristics (e.g., stage of breast cancer, prevalence of sarcopenia, sample size, participant age), measurement methods and criteria of sarcopenia, length of follow-up, and study quality were extracted from the included studies. The investigators cross-checked all extracted data at every step, and any disagreements were dealt with by discussion until a consensus was reached.

### Assessment of bias risk

Two independent reviewers (YZY, WWZ) assessed the risk of bias according to the Newcastle Ottawa Scale (NOS) [22]. The NOS includes six aspects, and the scale’s highest possible total score is 9 points. The following NOS information was used: (1) representativeness of the exposed cohort, (2) comparability of group, (3) blinding of investigators who measured outcomes, (4) time and completeness of follow-up, (5) contamination bias, and (6) other potential sources of bias. We regarded a total score of ≥5 points as high-quality research.

### Statistical analysis

Two authors (XMZ, YZY) analyzed the data independently using the software STATA version 14.0 (Stata Corp., College Station, TX, USA). Hazard ratios (HRs), and their 95% CIs of mortality for sarcopenic compared with non-sarcopenic participants were extracted from the studies that were included for meta-analysis. We also performed subgroup analyses according to stage of breast cancer, participant age, and length of follow-up if there was more than one study in a subgroup. The statistical heterogeneity of the included studies was examined with Cochran’s Q statistic using chi-square and *I*^2^ statistics, and we defined the cut-off *I*^2^ values of 25, 50, and 75% as low, moderate, and high heterogeneity, respectively. We decided to use a random-effects model based on heterogeneity when it was ≥50% or the *p*-value of the test of heterogeneity was less than 0.05. Otherwise, the fixed-effects model was used. We also conducted a publication bias and sensitivity analysis to test the stability of the meta-analysis, and the results were illustrated using forest plots.

In addition, in order to evaluate the reliability of the study results, we performed a trial sequential analysis (TSA) on all-cause mortality with a two-side *α* of 5% and a power of 90%. We assumed that breast cancer patients with sarcopenia would be linked with an at least 20% relative risk reduction in all-cause mortality.

### Patient and public involvement

Patients or members of the public were not involved in the study.

## Results

### Search results

**Fig. 1:**
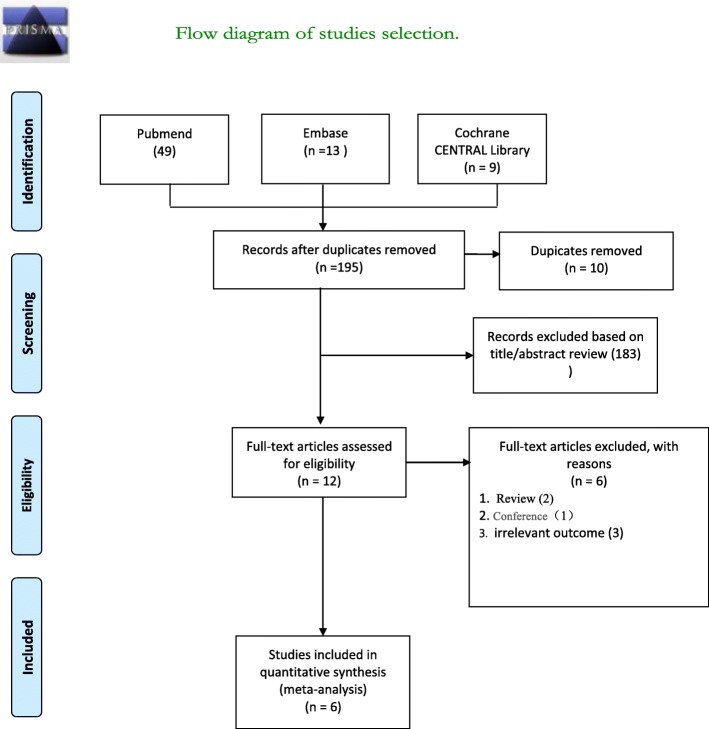
Flow diagram of studies selection

To start out, a total of 195 articles were confirmed by our literature search strategy. After removing 10 duplicates, 185 articles were screened for title and abstract. A total of 12 publications remained for further consideration by full-text review. Of these articles, three were removed because they were non-cohort studies (e.g., review articles, conference abstracts), and three studies were removed due to irrelevant outcomes or no clear definition of sarcopenia. These studies were identified based on the predefined inclusion and exclusion criteria in the meta-analysis, resulting in a total of six articles (Fig. 1).

### Quality assessment

The results of quality assessment are shown in Table 1 with detailed designation of the methodological quality evaluation using NOS. Our results indicated that the scores ranged from 6 to 9, and five studies reported scores ≥7.

**Table 1:** Result of the Newcastle-Ottawa scale quality assessment

Newcastle-Ottawa scale	Selection (1)				Comparability (2)	Outcome(3)			Total
	Representativeness of the exposed cohort	Selection of the non-exposed cohort	Ascertainment of exposure	Demonstration that outcome of interest was not present at start of study	Comparability of cohorts on the basis of the design or analysis	Assessment of outcome	Was follow-up long enough for outcome to occur	Adequacy of follow up of cohorts	
Shachar	1	1	1	1	0	1	0	1	6
Villasenor	1	1	1	1	1	1	1	1	8
Rier	1	1	1	1	1	1	0	1	7
Caan	1	1	1	1	2	1	1	1	9
Deluche	1	1	1	1	1	1	0	1	7
Song	1	1	1	1	1	1	1	1	8

### Prevalence of sarcopenia in female breast cancer patients and participant characteristics

**Fig. 2:**
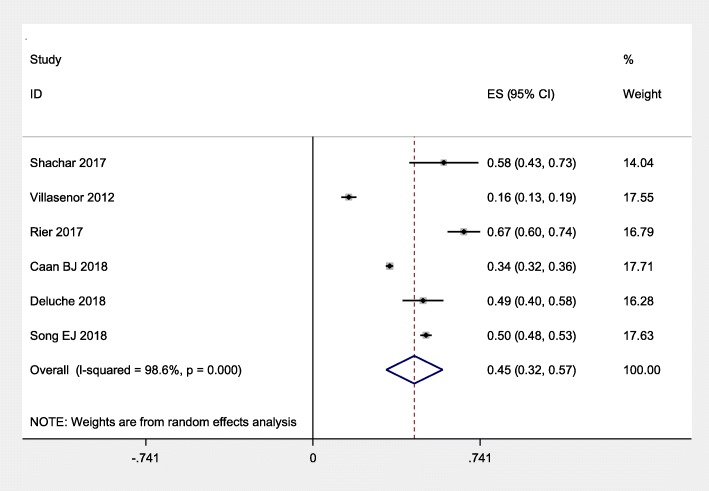
Pooled prevalence of sarcopenia among women with breast cancer

Table 2 displays the characteristics of the six studies, with 5497 participants, that were included. In all, the overall prevalence of sarcopenia was 45% [95% CI: 32–57%; I^2^ = 98.6%, *P* = 0.000] (Fig. 2). There were three studies conducted in the U.S. [15, 16, 19], one in France [17] and one study in Korea [18] and one in the Netherlands [18]. The mean age in all the studies ranged from 46 to 79.1 years old. All the studies considered all-cause mortality as the clinical outcome. There were two different stages of breast cancer examined, with four studies concentrating on patients with non-metastatic breast cancer [15, 17, 19, 20] and two others focusing on metastatic breast cancer [16, 18]. The largest study consisted of 3241 individuals [15], whereas the smallest cohort had only 40 individuals [16]. The length of follow-up varied from 1.2 to 12 years (Table 2).

**Table 2:** Summary of Included Studies on sarcopenia Associated with All-cause Mortality among breast cancer

Author	Year	Country	Disease stage	Sample	Age	“Sarcopenia Criteria”	Prevalence	Method	Follow-up	Outcome
Shachar	2017	USA	Metastatic	40	55 (11.7)	SMI derived from L3 muscle/height^2^ SMI < 41 cm^2^/m^2^	58.0%	CT	1.9 years	mortality
Villasenor	2012	USA	Non-Metastatic	471	79.17 (7.99)	ALM < 5.45 kg/m^2^	15.9%	DXA	9.2 years	mortality
Rier	2017	Netherlands	Metastatic	166	58.8 (11.3)	SMI derived from L3 muscle/height^2^ SMI < 41 cm^2^/m^2^	66.9%	CT	1.5 years	mortality
Caan	2018	USA	Non-metastatic	3241	54.1 (11.8)	SMI derived from L3 muscle mass/height^2^ SMI < 40 cm^2^/m^2^	34%	CT	12 years	mortality
Deluche	2018	France	Non-metastatic	119	56.0	SMI derived from L3 muscle/height^2^ SMI < 41 cm^2^/m^2^	48.8%	CT	4.36 years	mortality
Song	2018	Korea	Non-metastatic	1460	46.0	Skeletal muscle volume derived from L3 muscle. Sarcopenia was defined as less than the median muscle volume	50.0%	CT	8.07 years	mortality

*SMI* Skeletal Muscle Index, SMI < 41.0 cm2/m2 to determine sarcopenia

*ALM* Appendicular lean mass, sarcopenia was defined as two standard deviations below the young healthy adult female mean of appendicular lean mass (ALM) divided by height squared (< 5.45 kg/m2)

### Methods used to screen for sarcopenia in female breast cancer patients

Four studies used the most common method of SMI, which use CT scan at lumbar 3 to define sarcopenia [15–18], whereas one study used volume of skeletal muscle [20]. In addition, one study used dual X-ray absorptiometry scans to measure appendicular lean mass. The criterion used to define sarcopenia was two standard deviations below the young healthy adult female mean of appendicular lean mass (ALM) divided by height squared (< 5.45 kg/m^2^) [19].

### Sarcopenia as an independent predictor of all-cause mortality in female patients with breast cancer

**Fig. 3:**
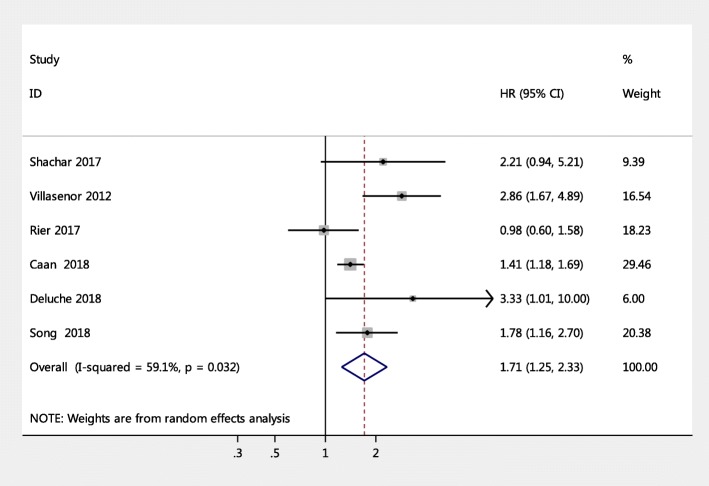
Meta-analysis of the association between sarcopenia and mortality among women with breast cancer

The pooled results showed that female breast cancer patients with sarcopenia had a significantly higher risk of all-cause mortality (pooled HR = 1.71, 95% CI = 1.25, 2.33, *p* < 0.001) versus participants without sarcopenia, indicating that sarcopenia significantly increases the risk of mortality for female breast cancer patients (Fig. 3), whereas there was significant heterogeneity between these studies (Q-value = 12.22, degree of freedom = 5, I^2^ = 59.1%, *P* = 0.032). TSA for all-cause mortality found that the required sample size was 4823 and that the Z line had crossed both information size and conventional boundaries, indicating that the association of sarcopenia and all-cause mortality was reliable and robust (Supplement Figure 1).

### Subgroup analysis of sarcopenia for all-cause mortality in female breast cancer patients

**Fig. 4:**
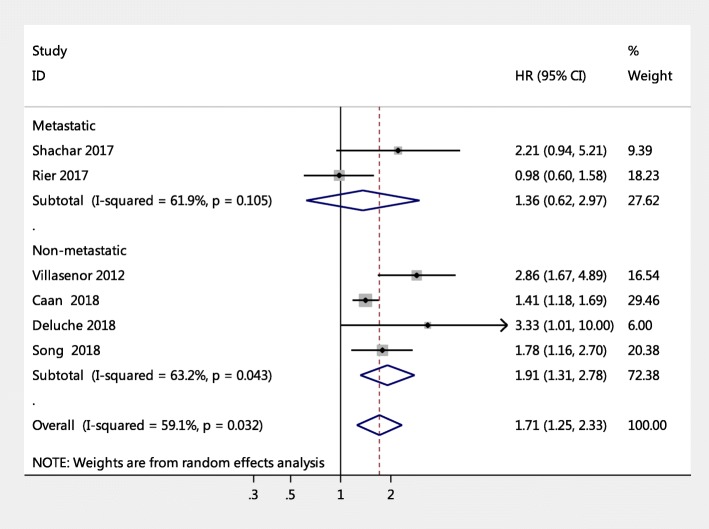
Subgroup meta-analysis of the association between sarcopenia and mortality among women with breast cancer by disease stage

**Fig. 5:**
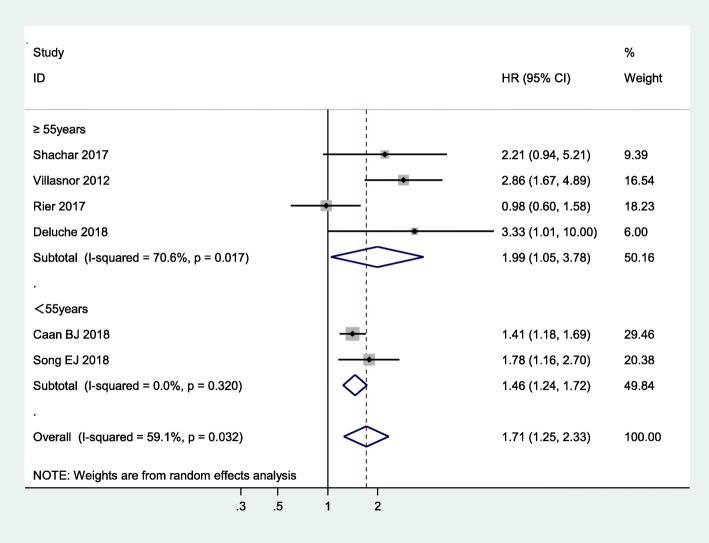
Subgroup meta-analysis of the association between sarcopenia and mortality among women with breast cancer by age group

**Fig. 6:**
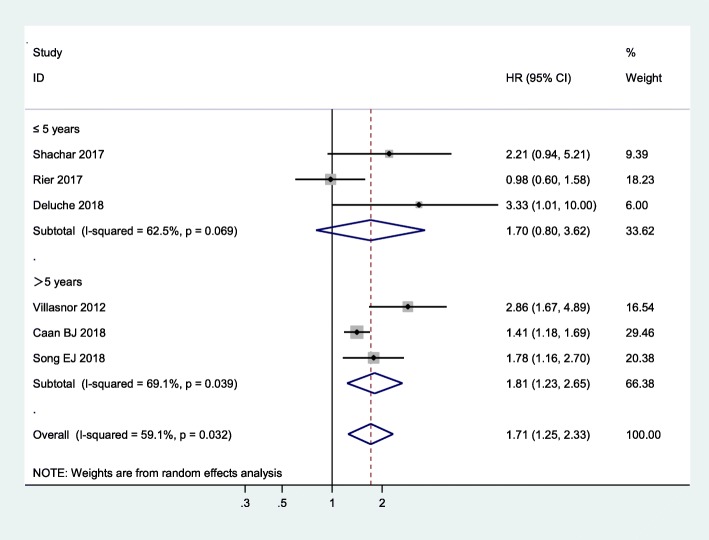
Subgroup meta-analysis of the association between sarcopenia and mortality among women with breast cancer according to length of follow-up

Subgroup analysis in terms of breast cancer stage showed that sarcopenic individuals with non-metastatic cancer face an augmented risk of mortality versus non-sarcopenic individuals (pooled HR = 1.91, 95% CI = 1.31, 2.78, *p* = 0.001, I^2^ = 63.2%, *P* = 0.043), whereas this association was not significant in sarcopenic individuals with metastatic breast cancer (pooled HR = 1.36, 95% CI = 0.62, 2.97; I^2^ = 61.9%, *P* = 0.105), as shown in Fig. 4. In addition, age subgroup analysis found that breast cancer patients with sarcopenia had an increased risk of mortality independent of age group, but participants aged 55 years and older with sarcopenia had a higher risk of mortality than participants younger than 55 years with sarcopenia (pooled HR = 1.99, 95% CI: 1.05–3.78; I^2^ = 70.6%, *P* = 0.017 versus pooled HR = 1.46, 95% CI: 1.24–1.72; I^2^ = 0%, *P* = 0.320) (Fig. 5). Other subgroup analyses were conducted according to length of follow-up (> 5 years versus ≤5 years): Fig. 6 shows that with a follow-up period of more than five years, there was a significantly higher risk of mortality in female breast cancer patients (pooled HR = 1.81, 95% CI: 1.23–2.65), but this association was not significant with a follow-up period of less than five years (pooled HR = 1.70, 95% CI: 0.80–3.62).

### Publication bias assessment

There is no significant publication bias included in this meta-analysis (Begg’s test: *P* = 0.260 and Egger’s = 0.157) as shown in Supplemental Figure 2.

### Sensitivity analysis of all studies

A sensitivity analysis of sarcopenia and mortality was conducted by omitting one study each time and pooling the others to determine which study would influence the pooled effect. There were no statistically significant changes among these studies, as shown in Supplemental Figure 3.

## Discussion

This study examined the association between sarcopenia and mortality in female breast cancer patients. The findings showed that breast cancer patients with sarcopenia had a 71% increased risk of mortality compared to patients without sarcopenia but have high heterogeneity. To the best of our knowledge, this is the first meta-analysis to systematically investigate the relationship between sarcopenia and all-cause mortality in female breast cancer patients. Our study indicated that screening female breast cancer patients for sarcopenia is crucial, because it may be a prognostic factor for female breast cancer patients.

The association between sarcopenia and mortality has been explored in many different populations, ranging from community-dwelling older adults [23] to nursing home residents [24]. Recently, a number of studies have found that sarcopenia can increase the risk of mortality among patients with certain types of cancer, such as lung cancer [25], gastric cancer [26], and colorectal cancer [27], indicating that sarcopenia can be a predictive factor in cancer patients. This study has an important implication for medical personnel. First, for patients with early-stage breast cancer, screening for sarcopenia by means of simple CT images or dual-energy X-rays can provide information to medical personnel regarding when to initiate interventions so as to delay or even prevent sarcopenia and thus promote patients’ survival. Several studies have reported that physical training (e.g. aerobic or resistance exercises) [28, 29] and nutritional supplements (e.g. vitamin D or omega-3 fatty acid dietary supplements) [30, 31] can prevent the loss of muscle mass. Furthermore, our study found that the prevalence of sarcopenia in breast cancer patients was 45% [95% CI: 32–57], which was higher than in community-dwelling older adults [32]. Considering this together with the results of previous studies substantiating that sarcopenia can increase the risk of negative clinical outcomes [33, 34], it is recommended that assessment of sarcopenia should be incorporated as part of the routine clinical assessment for patients with breast cancer, particularly for those who are in the early stage.

In addition, metastatic breast cancer patients usually receive chemotherapy or radiotherapy to increase their overall likelihood of survival. However, they are susceptible to drug side effects and other complications such as malnutrition and cachexia [35]. How to optimize a chemotherapy regimen for metastatic breast cancer patients remains a long-standing dilemma in clinical practice. Traditionally, physicians calculate the dose of chemotherapy according to the body surface area [36]. Recently, some studies have reported that breast cancer patients with sarcopenia have greater risk of grade 3–4 toxicity and of suffering from a number of adverse effects than non-sarcopenic breast cancer patients [16]. This indicates that sarcopenic breast cancer patients are more vulnerable to the side effects of chemotherapy. Therefore, screening for sarcopenia, particularly among metastatic breast cancer patients, becomes important for determining chemotherapy dosage.

The underlying mechanism that causes sarcopenia to increase the risk of all-cause mortality among breast cancer patients is more complicated. These factors may explain the relatively strong correlation between sarcopenia and mortality. First, the main feature of sarcopenia is muscle loss, which is a result of an imbalance between the pathways of synthesis and degradation of proteins, leading to an increase in muscle cell apoptosis and a decline in regenerative capacity [37]. That muscle loss increases the risk of mortality has been confirmed in several previous studies [38, 39]. Second, there is more evidence showing that muscle atrophy is associated with immune pathways and inflammation [39]. Previous studies have found that lower levels of muscle mass are distinctly associated with high neutrophil to lymphocyte ratios, which are markers of systemic inflammation, which increases mortality [40]. Third, sarcopenia is linked to proteolytic cascades, for instance the tumor necrosis factor (TNF-α) [41], which have been demonstrated to promote tumor migration and invasion and are associated with a deterioration in breast cancer prognoses [42]. Last but not least, sarcopenia is a geriatric syndrome rather than a disease, involving nervous system alterations as well as nutritional, hormonal, immunological, pro-inflammatory cytokines, aging and physical activity changes [43]. The mechanism behind how sarcopenia leads to increased risk of mortality is very complex and requires more scientific research.

We conducted a subgroup analysis by disease stage and found that the presence of sarcopenia with non-metastatic breast cancer increased the risk of mortality compared to non-metastatic breast cancer without sarcopenia. However, the findings of this meta-analysis did not show an increased risk of mortality among metastatic breast cancer patients. It is acknowledged that there is high heterogeneity in each age group. We were particularly surprised by this result, as a previous study had confirmed that sarcopenia increased the risk of mortality in patients with metastatic solid tumors [14]. Possible reasons could be that only two studies explored the relationship between sarcopenia and mortality in metastatic breast cancer patients. It is acknowledged that there were only 206 cases in the two studies of metastatic breast cancer patients, which means that some selective bias could have existed. Hence, it is possible that the number of studies included for analysis was too small to produce a significant result. Therefore, further studies on this issue should be conducted to clarify this unexpected result.

Age subgroup analysis found that participants aged 55 years and older with sarcopenia had a higher risk of mortality than participants younger than 55 years with sarcopenia. The results of this study suggest that aging could possibly play an important role in disease prognosis. According to some studies, the prevalence of sarcopenia increases as people age [44], while aging accelerates the process of sarcopenia [45]. Therefore, physicians need to screen breast cancer patients for sarcopenia earlier and undertake interventions to treat breast cancer patients with sarcopenia.

Our study has both strengths and limitations. One strength was that we conducted appropriate statistical analysis and performed comprehensive sensitivity and publication bias analysis. In addition, to our knowledge, this is the first meta-analysis to explore the relationship between sarcopenia and all-cause mortality in breast cancer patients. However, our systematic review and meta-analysis have some limitations. These limitations include the inclusion of studies that are observational and lack randomized controlled trials, which might include confounding factors that might influence the result. Furthermore, the number of studies included is small, which means that we could not perform certain subgroup analyses, such as subgroup analysis of sarcopenia measurement. In addition, it is acknowledged that breast cancer patients with sarcopenia may also have cachexia, which can affect the degree of muscle function. Unfortunately, all six studies included in this review did not take cachexia into account. Finally, the cut-off values for defining sarcopenia were different. One study used median muscle volume to dichotomize patients as having sarcopenia or not, whereas four studies used SMI < 41.0 cm^2^/m^2^ to determine sarcopenia, which means that there could be an overestimation or underestimation of the effects of sarcopenia.

## Conclusions

The prevalence of sarcopenia is high among women with breast cancer. Our study found that sarcopenia indicates a high risk of mortality among women with early-stage breast cancer. More research into the effects of specific interventions, such as physical exercise and supplemental nutrition, aimed at treating sarcopenia need to be conducted in the future.

## Supplementary information

**Additional file 1 Supplement 1**. Trial sequential analysis (TSA) of all-cause mortality.

**Additional file 2 Supplement 1**. Funnel plot of the meta-analysis.

**Additional file 3 Supplement 2.** Sensitivity analysis of all studies.

**Additional file 4.** The search strategy for the PubMed database.

## Data Availability

The datasets supporting the conclusion of this article are available in the electronic databases (PubMed, EMBASE, and the Cochrane Library).

## Abbreviations

BIA: Bioelectrical impedance analysis
CT: Computed tomography
DEXA: Dual-energy X-ray absorptiometry
HRs: Hazard ratios
Mesh: Medical subject headings
MRI: Magnetic resonance imaging
NOS: Newcastle Ottawa Scale
OS: Overall survival

## References

[1] Torre LA, Bray F, Siegel RL, Ferlay J, Lortet-Tieulent J, Jemal A (2015). Global cancer statistics, 2012. CA Cancer J Clin.

[2] Bray F, Ferlay J, Soerjomataram I, Siegel RL, Torre LA, Jemal A (2018). Global cancer statistics 2018: GLOBOCAN estimates of incidence and mortality worldwide for 36 cancers in 185 countries. CA Cancer J Clin.

[3] Visser LL, Groen EJ, van Leeuwen FE, Lips EH, Schmidt MK, Wesseling J (2019). Predictors of an invasive breast Cancer recurrence after DCIS: a systematic review and meta-analyses. Cancer Epidemiol Biomarkers Prev.

[4] Argiles JM, Busquets S, Stemmler B, Lopez-Soriano FJ (2014). Cancer cachexia: understanding the molecular basis. Nat Rev Cancer.

[5] Fearon K, Strasser F, Anker SD, Bosaeus I, Bruera E, Fainsinger RL, Jatoi A, Loprinzi C, MacDonald N, Mantovani G (2011). Definition and classification of cancer cachexia: an international consensus. Lancet Oncol.

[6] da Rocha IMG, Marcadenti A, de Medeiros GOC, Bezerra RA, Rego JFM, Gonzalez MC, Fayh APT (2019). Is cachexia associated with chemotherapy toxicities in gastrointestinal cancer patients? A prospective study. J Cachexia Sarcopenia Muscle.

[7] Han JS, Ryu H, Park IJ, Kim KW, Shin Y, Kim SO, Lim SB, Kim CW, Yoon YS, Lee JL, et al. Association of Body Composition with long-term survival in non-metastatic rectal Cancer patients. Cancer Res Treat. 2019. 10.4143/crt.2019.249. 10.4143/crt.2019.249 PMC7176960 31801316

[8] Cruz-Jentoft Alfonso J, Bahat Gϋlistan, Bauer Jϋrgen, Boirie Yves, Bruyère Olivier, Cederholm Tommy, Cooper Cyrus, Landi Francesco, Rolland Yves, Sayer Avan Aihie, Schneider Stéphane M, Sieber Cornel C, Topinkova Eva, Vandewoude Maurits, Visser Marjolein, Zamboni Mauro (2019). Sarcopenia: revised European consensus on definition and diagnosis. Age and Ageing.

[9] Cruz-Jentoft AJ, Sayer AA (2019). Sarcopenia. Lancet.

[10] Pamoukdjian F, Bouillet T, Levy V, Soussan M, Zelek L, Paillaud E (2018). Prevalence and predictive value of pre-therapeutic sarcopenia in cancer patients: a systematic review. Clin Nutr.

[11] Cornet M, Lim C, Salloum C, Lazzati A, Compagnon P, Pascal G, Azoulay D (2015). Prognostic value of sarcopenia in liver surgery. J Visc Surg.

[12] Prado CM, Baracos VE, McCargar LJ, Reiman T, Mourtzakis M, Tonkin K, Mackey JR, Koski S, Pituskin E, Sawyer MB (2009). Sarcopenia as a determinant of chemotherapy toxicity and time to tumor progression in metastatic breast cancer patients receiving capecitabine treatment. Clin cancer Res.

[13] Ganju RG, Morse R, Hoover A, TenNapel M, Lominska CE (2019). The impact of sarcopenia on tolerance of radiation and outcome in patients with head and neck cancer receiving chemoradiation. Radiother Oncol.

[14] Shachar SS, Williams GR, Muss HB, Nishijima TF (2016). Prognostic value of sarcopenia in adults with solid tumours: a meta-analysis and systematic review. Eur J Cancer.

[15] Caan BJ, Cespedes Feliciano EM, Prado CM, Alexeeff S, Kroenke CH, Bradshaw P, Quesenberry CP, Weltzien EK, Castillo AL, Olobatuyi TA (2018). Association of muscle and adiposity measured by computed tomography with survival in patients with nonmetastatic breast cancer. JAMA Oncol.

[16] Shachar SS, Deal AM, Weinberg M, Nyrop KA, Williams GR, Nishijima TF, Benbow JM, Muss HB (2017). Skeletal muscle measures as predictors of toxicity, hospitalization, and survival in patients with metastatic breast cancer receiving taxane-based chemotherapy. Clin Cancer Res.

[17] Deluche E, Leobon S, Desport JC, Venat-Bouvet L, Usseglio J, Tubiana-Mathieu N (2018). Impact of body composition on outcome in patients with early breast cancer. Support Care Cancer.

[18] Rier HN, Jager A, Sleijfer S, van Rosmalen J, Kock M, Levin MD (2017). Low muscle attenuation is a prognostic factor for survival in metastatic breast cancer patients treated with first line palliative chemotherapy. Breast.

[19] Villaseñor A, Ballard-Barbash R, Baumgartner K, Baumgartner R, Bernstein L, McTiernan A, Neuhouser ML (2012). Prevalence and prognostic effect of sarcopenia in breast cancer survivors: the HEAL study. J Cancer Surviv.

[20] Song EJ, Lee CW, Jung SY, Kim BN, Lee KS, Lee S, Kang HS, Park IH, Lee MH, Kim YJ (2018). Prognostic impact of skeletal muscle volume derived from cross-sectional computed tomography images in breast cancer. Breast Cancer Res Treat.

[21] Rossi F, Valdora F, Bignotti B, Torri L, Succio G, Tagliafico AS (2019). Evaluation of body computed tomography-determined sarcopenia in breast cancer patients and clinical outcomes: a systematic review. Cancer Treat Res Commun.

[22] Higgins JP, Thompson SG, Deeks JJ, Altman DG (2003). Measuring inconsistency in meta-analyses. BMJ.

[23] Liu P, Hao Q, Hai S, Wang H, Cao L, Dong B (2017). Sarcopenia as a predictor of all-cause mortality among community-dwelling older people: a systematic review and meta-analysis. Maturitas.

[24] Zhang X, Wang C, Dou Q, Zhang W, Yang Y, Xie X (2018). Sarcopenia as a predictor of all-cause mortality among older nursing home residents: a systematic review and meta-analysis. BMJ Open.

[25] Deng HY, Hou L, Zha P, Huang KL, Peng L (2019). Sarcopenia is an independent unfavorable prognostic factor of non-small cell lung cancer after surgical resection: a comprehensive systematic review and meta-analysis. Eur J Surg Oncol.

[26] Kamarajah SK, Bundred J, Tan BHL (2019). Body composition assessment and sarcopenia in patients with gastric cancer: a systematic review and meta-analysis. Gastric Cancer.

[27] Sun G, Li Y, Peng Y, Lu D, Zhang F, Cui X, Zhang Q, Li Z (2018). Can sarcopenia be a predictor of prognosis for patients with non-metastatic colorectal cancer? A systematic review and meta-analysis. Int J Color Dis.

[28] Adams SC, Segal RJ, McKenzie DC, Vallerand JR, Morielli AR, Mackey JR, Gelmon K, Friedenreich CM, Reid RD, Courneya KS (2016). Impact of resistance and aerobic exercise on sarcopenia and dynapenia in breast cancer patients receiving adjuvant chemotherapy: a multicenter randomized controlled trial. Breast Cancer Res Treat.

[29] Dieli-Conwright CM, Courneya KS, Demark-Wahnefried W, Sami N, Lee K, Buchanan TA, Spicer DV, Tripathy D, Bernstein L, Mortimer JE (2018). Effects of aerobic and resistance exercise on metabolic syndrome, sarcopenic obesity, and circulating biomarkers in overweight or obese survivors of breast cancer: a randomized controlled trial. J Clin Oncol Off J Am Soc Clin Oncol.

[30] Kung T, Springer J, Doehner W, Anker SD, von Haehling S (2010). Novel treatment approaches to cachexia and sarcopenia: highlights from the 5th Cachexia conference. Expert Opin Investig Drugs.

[31] Di Girolamo FG, Situlin R, Mazzucco S, Valentini R, Toigo G, Biolo G (2014). Omega-3 fatty acids and protein metabolism: enhancement of anabolic interventions for sarcopenia. Curr Opin Clin Nutr Metab Care.

[32] Mayhew AJ, Amog K, Phillips S, Parise G, McNicholas PD, de Souza RJ, Thabane L, Raina P (2019). The prevalence of sarcopenia in community-dwelling older adults, an exploration of differences between studies and within definitions: a systematic review and meta-analyses. Age Ageing.

[33] Zhang X, Huang P, Dou Q, Wang C, Zhang W, Yang Y, Wang J, Xie X, Zhou J, Zeng Y (2019). Falls among older adults with sarcopenia dwelling in nursing home or community: a meta-analysis. Clin Nutr.

[34] Beaudart C, Zaaria M, Pasleau F, Reginster JY, Bruyere O (2017). Health outcomes of sarcopenia: a systematic review and meta-analysis. PLoS One.

[35] Maughan KL, Lutterbie MA, Ham PS (2010). Treatment of breast cancer. Am Fam Physician.

[36] Du Bois D, Du Bois EF (1989). A formula to estimate the approximate surface area if height and weight be known. 1916. Nutr.

[37] Argiles JM, Busquets S, Stemmler B, Lopez-Soriano FJ (2015). Cachexia and sarcopenia: mechanisms and potential targets for intervention. Curr Opin Pharmacol.

[38] Wang H, Hai S, Liu Y, Liu Y, Dong B (2019). Skeletal muscle mass as a mortality predictor among nonagenarians and centenarians: a prospective cohort study. Sci Rep.

[39] Weijs PJ, Looijaard WG, Dekker IM, Stapel SN, Girbes AR, Oudemans-van Straaten HM, Beishuizen A (2014). Low skeletal muscle area is a risk factor for mortality in mechanically ventilated critically ill patients. Crit Care.

[40] Feliciano EMC, Kroenke CH, Meyerhardt JA, Prado CM, Bradshaw PT, Kwan ML, Xiao J, Alexeeff S, Corley D, Weltzien E (2017). Association of systemic inflammation and sarcopenia with survival in nonmetastatic colorectal cancer: results from the C SCANS study. JAMA Oncol.

[41] Bian AL, Hu HY, Rong YD, Wang J, Wang JX, Zhou XZ (2017). A study on relationship between elderly sarcopenia and inflammatory factors IL-6 and TNF-alpha. Eur J Med Res.

[42] Liu D, Wang X, Chen Z (2016). Tumor necrosis factor-alpha, a regulator and therapeutic agent on breast cancer. Curr Pharm Biotechnol.

[43] Narici MV, Maffulli N (2010). Sarcopenia: characteristics, mechanisms and functional significance. Br Med Bull.

[44] Bianchi L, Abete P, Bellelli G, Bo M, Cherubini A, Corica F, Di Bari M, Maggio M, Manca GM, Rizzo MR (2017). Prevalence and clinical correlates of sarcopenia, identified according to the EWGSOP definition and diagnostic algorithm, in hospitalized older people: the GLISTEN study. J Gerontol A Biol Sci Med Sci.

[45] Waltz TB, Fivenson EM, Morevati M, Li C, Becker KG, Bohr VA, Fang EF (2018). Sarcopenia, aging and prospective interventional strategies. Curr Med Chem.

