# Supplementary material for: Sarcopenia as a predictor of mortality in women with breast cancer: a meta-analysis and systematic review
Source: BMC Cancer. 2020 Mar 4;20:172. doi: 10.1186/s12885-020-6645-6 (PMC7057618; doi:10.1186/s12885-020-6645-6)
Supplement: Supplementary file 1 — Additional file 1 Supplement 1. Trial sequential analysis (TSA) of all-cause mortality. [file 12885_2020_6645_MOESM1_ESM.doc]

**Supplement 1**: Funnel plot of the meta-analysis.
