# Supplementary material for: Sarcopenia as a predictor of mortality in women with breast cancer: a meta-analysis and systematic review
Source: BMC Cancer. 2020 Mar 4;20:172. doi: 10.1186/s12885-020-6645-6 (PMC7057618; doi:10.1186/s12885-020-6645-6)
Supplement: Supplementary file 2 — Additional file 2 Supplement 1. Funnel plot of the meta-analysis. [file 12885_2020_6645_MOESM2_ESM.doc]

**Supplement 2.** Sensitivity analysis of all studies.
