# Supplementary figures and images for: Sarcopenia as a predictor of mortality in women with breast cancer: a meta-analysis and systematic review
Source: BMC Cancer. 2020 Mar 4;20:172. doi: 10.1186/s12885-020-6645-6 (PMC7057618; doi:10.1186/s12885-020-6645-6)

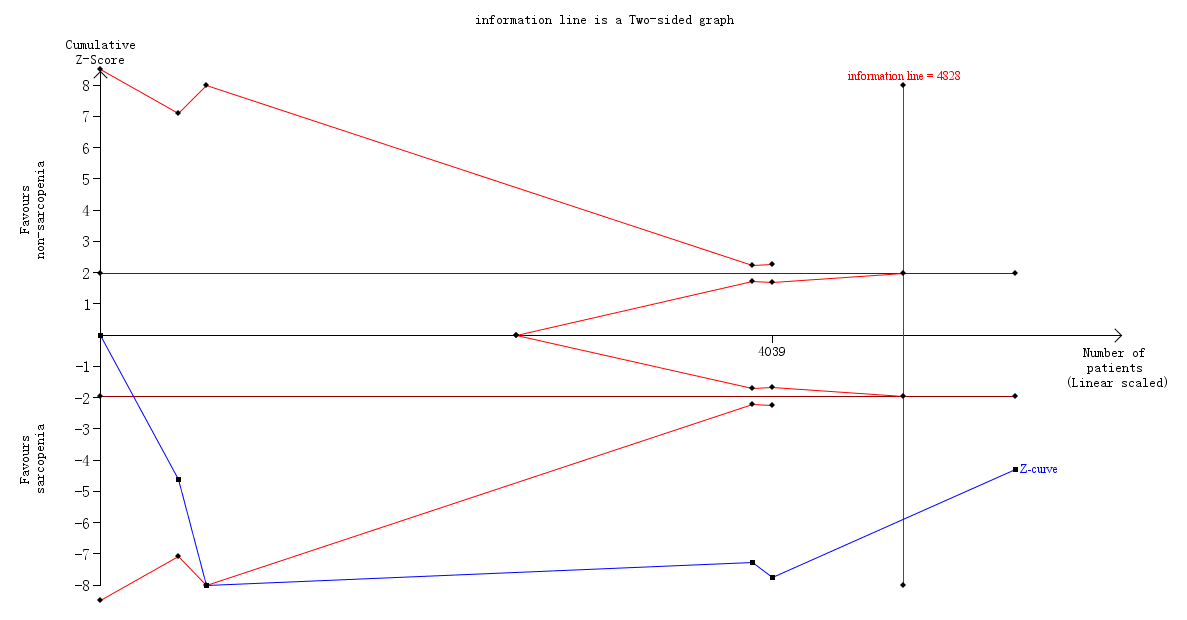


**Supplement 1**: Trial sequential analysis (TSA) of all-cause mortality

Supplement: Supplementary file 3 — Additional file 3 Supplement 2. Sensitivity analysis of all studies. [file 12885_2020_6645_MOESM3_ESM.doc]
