# Supplementary material for: Sarcopenia as a predictor of mortality in women with breast cancer: a meta-analysis and systematic review
Source: BMC Cancer. 2020 Mar 4;20:172. doi: 10.1186/s12885-020-6645-6 (PMC7057618; doi:10.1186/s12885-020-6645-6)
Supplement: Supplementary file 4 — Additional file 4. The search strategy for the PubMed database. [file 12885_2020_6645_MOESM4_ESM.docx]

**The search strategy for the PubMed database**

#1 :(("breast tumor") OR "breast cancer") OR "Breast Neoplasms"[Mesh])

#2: ((preSarcopenia) OR Sarcopenia*) OR "Sarcopenia"[Mesh]

#3: #2 and #1
